# Psychological trauma and posttraumatic stress disorder: risk factors and associations with birth outcomes in the Drakenstein Child Health Study

**DOI:** 10.3402/ejpt.v7.28720

**Published:** 2016-02-12

**Authors:** Nastassja Koen, Kirsty Brittain, Kirsten A. Donald, Whitney Barnett, Sheri Koopowitz, Karen Maré, Heather J. Zar, Dan J. Stein

**Affiliations:** 1 Department of Psychiatry & Mental Health, University of Cape Town, Cape Town, South Africa; 2 Medical Research Council (MRC) Unit on Anxiety & Stress Disorders, Cape Town, South Africa; 3 Department of Paediatrics and Child Health, Red Cross War Memorial Children's Hospital, University of Cape Town and Medical Research Council (MRC) Unit on Child & Adolescent Health, Cape Town, South Africa; 4 Division of Developmental Paediatrics, Department of Paediatrics & Child Health, Red Cross War Memorial Children's Hospital, University of Cape Town, Cape Town, South Africa

**Keywords:** Trauma, PTSD, pregnancy, intergenerational, birth cohort, birth outcomes, South Africa

## Abstract

**Background:**

Prenatal and peripartum trauma may be associated with poor maternal–fetal outcomes. However, relatively few data on these associations exist from low-middle income countries, and populations in transition.

**Objective:**

We investigated the prevalence and risk factors for maternal trauma and posttraumatic stress disorder (PTSD), and their association with adverse birth outcomes in the Drakenstein Child Health Study, a South African birth cohort study.

**Methods:**

Pregnant women were recruited from two clinics in a peri-urban community outside Cape Town. Trauma exposure and PTSD were assessed using diagnostic interviews; validated self-report questionnaires measured other psychosocial characteristics. Gestational age at delivery was calculated and birth outcomes were assessed by trained staff. Multiple logistic regression explored risk factors for trauma and PTSD; associations with birth outcomes were investigated using linear regression. Potential confounders included study site, socioeconomic status (SES), and depression.

**Results:**

A total of 544 mother–infant dyads were included. Lifetime trauma was reported in approximately two-thirds of mothers, with about a third exposed to past-year intimate partner violence (IPV). The prevalence of current/lifetime PTSD was 19%. In multiple logistic regression, recent life stressors were significantly associated with lifetime trauma, when controlling for SES, study site, and recent IPV. Childhood trauma and recent stressors were significantly associated with PTSD, controlling for SES and study site. While no association was observed between maternal PTSD and birth outcomes, maternal trauma was significantly associated with a 0.3 unit reduction (95% CI: 0.1; 0.5) in infant head-circumference-for-age z-scores (HCAZ scores) at birth in crude analysis, which remained significant when adjusted for study site and recent stressors in a multivariate regression model.

**Conclusions:**

In this exploratory study, maternal trauma and PTSD were found to be highly prevalent, and preliminary evidence suggested that trauma may adversely affect fetal growth, as measured by birth head circumference. However, these findings are limited by a number of methodological weaknesses, and further studies are required to extend findings and delineate causal links and mechanisms of association.

Exposure to psychological trauma is highly prevalent in South Africa. According to nationally representative data from the South African Stress and Health Study (SASH) (Williams et al., 2004), most individuals experience at least one traumatic event in their lifetimes, including criminal victimization, witnessing atrocities, and intimate partner violence (IPV) (Williams et al., 2007). Posttraumatic stress disorder (PTSD) is a debilitating stress-related psychiatric disorder affecting vulnerable individuals after trauma exposure and has a reported lifetime population prevalence of 2.3% among South Africans (Herman et al., 2009). A focus on PTSD is highly relevant in low-middle income (LMIC) settings, such as South Africa, given the high trauma burden and additive risk factor profile in this context.

Findings from the National Comorbidity Survey (Kessler, Chiu, Demler, & Walters, 2005; Kessler, Sonnega, Bromet, Hughes, & Nelson, 1995), a nationally representative study of 5,877 adults in the United States, indicated that women were overall more than twice as likely as men to develop lifetime PTSD (10.4% vs. 5.0%, respectively). Pregnant women and new mothers, in particular, comprise a highly vulnerable subgroup (e.g., Beck, Gable, Sakala, & Declercq, 2011; Söderquist, Wijma, Thorbert, & Wijma, 2009; Zaers, Waschke, & Ehlert, 2008). Prenatal trauma may place mothers at a high risk of developing PTSD and may result in a number of adverse maternal and fetal sequelae. For example, there is a strong body of evidence documenting the association between maternal trauma and related disorders, and delivery of an infant with low birthweight (LBW). Murphy, Schei, Myhr, and Du Mont (2001) provided meta-analytical evidence that physical, sexual, or emotional abuse during pregnancy may be part of a complex causative pathway contributing to low infant birthweight. In their prospective study of a cohort of 301 pregnant women from New Orleans and Baton Rouge who had been exposed to Hurricane Katrina, Xiong et al. (2008) reported that the frequency of LBW was notably higher among woman with high hurricane exposure versus those without high hurricane exposure (aOR 3.3; 95% CI: 1.13–9.89). The frequency of preterm birth was also found to be higher in the exposed group (aOR 2.3; 95% CI: 0.82–6.38). Similarly, in their prospective cohort study of 839 nulliparas in Michigan, USA, Seng, Low, Sperlich, Ronis, and Liberzon (2011) reported that infants born to women who had experienced PTSD during pregnancy had a mean birthweight which was 283 g less than those who had not developed PTSD following trauma exposure. Even the resilient (trauma-exposed) women were found to deliver infants with a mean birthweight which was 221 g less than that of the non-traumatized group. PTSD was also found to be associated with shorter gestation in this cohort. Adverse birth outcomes such as these are important risk factors for poor growth and neurodevelopment in infancy and childhood.

South Africa provides a unique context for further work in the field of psychological trauma exposure and consequent PTSD. First, most individuals experience multiple traumatic events across their lifespan. Second, gender- and pregnancy-specific trauma is rife, and likely to exert a range of adverse intergenerational effects. Third, ours is a population in transition. Despite a decline in recent years, the rate of urbanization and urban population growth in sub-Saharan Africa remains among the highest worldwide (Tacoli, McGranahan, & Satterthwaite, 2015). Furthermore, the number of lifetime immigrants in South Africa increased from approximately 800,000 pre-2001 to almost 1.5 million between 2001 and 2011 (Statistics SA, 2014). The Western Cape was found to have the second highest provincial net migration rate during this period. Female migrants in particular may be at increased risk of gender-specific trauma such as violence; inadequate access to basic infrastructure; and the burden of unpaid, unsupported, care work. The relationship between trauma or PTSD and adverse birth outcomes is also likely complex and may be influenced by a number of related sociodemographic and psychosocial risk factors that are prevalent in LMIC settings such as South Africa (see Kramer, 1987; Murphy et al., 2001; Paarlberg et al., 1999). Finally, despite the high burden of trauma and subsequent effects, there remains a relative dearth of data in this field from LMIC populations in transition such as ours. Thus, the current study sought to investigate the prevalence and risk factors for trauma and PTSD in a South African birth cohort study, and to explore associations with infant birth outcomes. We hypothesized that maternal trauma and PTSD would adversely affect infant birth outcomes, including weight-for-age z-score (WAZ), head-circumference-for-age z-score (HCAZ), size for gestational age, and gestational age at delivery.

## Methods

This study reports data from the Drakenstein Child Health Study (DCHS), a multidisciplinary birth cohort study investigating the determinants of child health over time (Stein et al., 2015; Zar, Barnett, Myer, Stein, & Nicol, 2014). This analysis includes a subsample of mothers enrolled into the cohort between March 2012 and October 2014.

### Participants

Pregnant women were recruited from two primary health care clinics, Mbekweni (serving a predominantly black African community) and TC Newman (serving a mixed race community), in a poor, peri-urban community outside Cape Town, South Africa. Mothers were enrolled in the DCHS at 20 to 28 weeks’ gestation and will be followed longitudinally throughout their pregnancy until 5 years postnatally. Exclusion criteria were individuals who were younger than 18 years, who did not attend study clinics for postnatal care (and thus could not be readily followed up), or those who were intending to move out of the district within 2 years after the infant's birth.

### Ethics

The DCHS was approved by the Faculty of Health Sciences human research ethics committees of the University of Cape Town (UCT) and Stellenbosch University in South Africa, and by the Western Cape Department of Health Provincial Research Committee. All study participants provided written informed consent.

Women were asked to complete a battery of self-report measures administered by on-site trained fieldworkers in the participants’ preferred language (English, Afrikaans, or isiXhosa). Translation of measures from English to Afrikaans and isiXhosa was done using standard forward- and back-translation processes (Smit, van den Berg, Bekker, Seedat, & Stein, 2006). A private consultation room was provided at each study site. Participants could choose not to answer certain questions and still remain in the study, as long as exposure and diagnostic status could be determined reasonably. Every effort was made to ensure a safe, confidential, and supportive environment.

### Measures

A number of reliable and validated questionnaires suitable for use in the South African context were administered to enrolled women at an antenatal clinic visit between 28 and 32 weeks’ gestation, as has been described elsewhere (Koen et al., 2014; Stein et al., 2015; Zar et al., 2014).

1. ***Maternal Sociodemographic Variables***—A questionnaire to assess socioeconomic status (SES) was adapted from the version used in the SASH (Myer, Stein, Grimsrud, Seedat, & Williams, 2008). Composite SES scores were calculated from this questionnaire from four sociodemographic variables, that is, educational attainment, employment status, household income, and assets and market access. Participants were then stratified into quartiles based on their relative SES score, that is, lowest, low-moderate, moderate-high, and highest SES. These quartiles were generated for the purposes of this study and represent an internal comparison for the study sample.
2. ***Psychosocial Risk Factors***—The World Mental Health Life Events Questionnaire (adapted from Myer et al., 2008) was used to assess exposure to stressful/negative life events during the past 12 months. This questionnaire includes certain items specifically defined as trauma in the DSM-5 (American Psychiatric Association [APA], 2013), that is, involving “death, threatened death, actual or threatened serious injury, or actual or threatened sexual violence,” as well as more general life stressors, such as job loss and robbery. The Beck Depression Inventory (BDI-II), a commonly used, reliable screen for depressive symptoms (Beck, Steer, & Brown, 1996; Beck, Steer, & Garbin, 1988; Beck, Ward, Mendelson, Mock, & Erbaugh, 1961), was used as the primary tool to assess for depression. The Edinburgh Postnatal Depression Rating Scale (EPDS) (Cox, Holden, & Sagovsky, 1987) is a 10-item self-report measure of recent depressive symptoms, which has shown good psychometric properties in validation studies (Eberhard-Gran et al., 2001). The SRQ-20 (Harding et al., 1980; Scholte, Verduin, van Lammeren, Rutayisire, & Kamperman, 2011) was used as a measure of psychological distress, and to supplement the findings of the depression screening tools. Substance misuse was assessed using the Alcohol, Smoking, and Substance Involvement Screening Test (ASSIST), a reliable, feasible, and validated questionnaire devised by the World Health Organization (WHO) (WHO ASSIST Working Group, 2002). The ASSIST comprises seven items assessing alcohol and other drug use across 10 categories, as well as an eighth item enquiring about a history of intravenous drug use.
3. ***Trauma Exposure and PTSD***—The Childhood Trauma Questionnaire (CTQ) (Bernstein et al., 1994) is a 28-item inventory assessing three domains of childhood abuse (sexual, physical, and emotional), and two domains of childhood neglect (physical and emotional), occurring at or before the age of 12 years. The Intimate Partner Violence Questionnaire was adapted from the WHO multicountry study (Jewkes, 2002) and the Women's Health Study (Zimbabwe) (Shamu, Abrahams, Temmerman, Musekiwa, & Zarowsky, 2011) and assessed lifetime and recent (past-year) exposure to emotional, physical, and sexual abuse. The Mini International Neuropsychiatric Interview (MINI) is an abridged version of the Structured Clinical Interview (SCID) for DSM-IV (Lecrubier et al., 1997; Sheehan et al., 1997, 1998). This clinician-administered interview was used to obtain a more detailed phenotypic description of trauma exposure (as defined by the DSM-5 criteria (APA, 2013)) and of lifetime PTSD in this study sample, from a number of antenatal and postnatal timepoints. Based on their responses to the MINI items, participants were categorized as having no trauma exposure, being trauma exposed with no PTSD, and being trauma exposed with PTSD.

Those women with a suspected high burden of psychiatric symptoms or stressors were referred to local routine service providers according to a structured standard operation procedure designed for the purposes of this study. In summary, participants with suspected psychopathology (as defined by the self-report tools) were detected on-site by trained fieldworkers, and ultimately referred to the study medical officer (MO). Following a psychiatric interview and risk assessment, the MO would then facilitate the most appropriate course of action for individual participants, including referral to emergency psychiatric services in Paarl. Educational pamphlets designed by the study team were made available to all study participants. These pamphlets included information on common mental health disorders, as well as contact details for freely accessible resources in the Drakenstein subdistrict.

### Birth outcomes

Anthropometric variables (weight, height, and head circumference) at birth were measured by clinical staff at Paarl Hospital and z-scores were calculated. LBW was defined as less than 2,500 g according to the WHO parameters (World Health Organization [WHO], 2014). Low WAZ (standard deviation score) was defined as a score less than or equal to 2 standard deviations below the mean weight-for-age (WFA) value (World Health Organization [WHO], 1995). Low HCAZ was similarly defined. Gestational age was recorded from early ultrasound measurements done during the second trimester (preferred method), inferred from maternal fundal height at enrollment or calculated from the self-reported date of last known menstrual period (Stein et al., 2015). Gestational age at delivery was then calculated in completed weeks of gestation, with infants categorized as small-for-gestational-age (SGA) or appropriate/large-for-gestational-age based on the revised Fenton preterm growth charts (Brittain et al., 2015; Fenton & Kim, 2013; Fenton et al., 2013). Prematurity was defined as birth before completion of 37 weeks’ gestation. For our purposes, most birth outcomes of interest (i.e., birthweight, WAZ scores, and HCAZ scores) were expressed as both dichotomous and continuous variables, while SGA and preterm delivery were dichotomized. The present analysis was restricted to live births only.

### Statistical analyses

All data were analyzed using Stata 12 (StataCorp Inc., College Station, TX, USA). Frequency distributions and medians (interquartile ranges [IQR]) were used to describe sociodemographic variables of interest (age, marital status, income); childhood and adult trauma exposure and stressful life events; PTSD, depression, and psychological distress; alcohol and substance use; and birth anthropometry/gestation. Variables significantly associated with lifetime maternal trauma exposure and, among trauma-exposed participants, with lifetime PTSD were identified using chi-square tests for categorical variables and Wilcoxon rank sum tests (Mann–Whitney tests) for non-normally distributed continuous variables. Odds ratios (OR) with 95% confidence intervals (CI) were calculated to determine the strength of these associations. The associations between PTSD, potential confounders, birth anthropometric outcomes (WAZ, HCAZ, SGA) and gestational age at delivery were then explored. The selection of relevant covariates was informed by model building based on directed acyclic graphs which were generated prior to conducting the analyses. This was in order to differentiate between potential confounders versus mediating variables. Variables significantly associated each with trauma exposure and with PTSD in bivariate analysis (at *p*<0.05) were included in regression models to examine independent predictors of decreased WAZ, decreased HCAZ, SGA, and shortened gestational age at delivery, respectively. Likelihood ratio tests were used to assess model fit.

## Results

### Maternal sociodemographic characteristics

Between March 2012 and October 2014, 1,047 mothers were enrolled in the DCHS. Those who had not yet given birth, and who had not yet completed the relevant ante- and postnatal assessments, were excluded from the present analysis. Data from an additional 14 women who had experienced antenatal losses or stillbirths during this time were also omitted. Thus, data from 544 mothers and 546 infants (including two sets of twins) were included in the final analysis.

Women had a median age of 26 years (IQR: 21.9; 31.3), with most (62%) being unmarried (Table 1). Although most women (89%) had received some high school education (Grades 8–12), the vast majority (80%) was unemployed. While the overall SES of this study sample was low (87% reported a household income of less than R5,000, approximately 400 USD, per month), a site-specific disparity was evident. Almost 50% of participants at Mbekweni reported an average household income of less than R1,000 (approximately 80 USD) per month, versus 35% at TC Newman. Notably, almost a third (32%) of the total study sample and more than half (55%) at Mbekweni reported being born outside of the Western Cape. Almost half (49%) of this study sample reported living in an urban setting, with 44% living in a township and only a minority (7%) residing in a rural area. Approximately 20% of mothers were HIV infected.

**Table 1:** Maternal demographic and psychosocial characteristics

Variable	Mbekweni—*n* (%)	TC Newman—*n* (%)	Total—*n* (%)
Number of mothers	309 (57)	235 (43)	544
Visit timepoint of MINI interview			
Antenatal	100 (32)	76 (32)	176 (32)
6–10 weeks	9 (3)	14 (6)	23 (4)
6 months	182 (59)	119 (51)	301 (55)
18 months	18 (6)	26 (11)	44 (8)
Ethnicity			
Black/African	305 (99)	1 (0.4)	306 (56)
Mixed race	4 (1)	232 (99)	236 (43)
Other	0 (0)	2 (0.9)	2 (0.4)
Migration			
Born in the Western Cape	140 (45)	228 (97)	368 (68)
Born outside of the Western Cape	169 (55)	7 (3)	176 (32)
Area of dwelling			
Urban	40 (13)	225 (96)	265 (49)
Rural	32 (10)	8 (3)	40 (7)
Township	237 (77)	2 (0.9)	239 (44)
Age at enrollment—median [IQR]	26.8 [22.1, 32.3]	25.0 [21.6, 29.7]	25.8 [21.9, 31.3]
Married/cohabiting	107 (35)	101 (43)	208 (38)
Educational achievement			
Primary	35 (11)	25 (11)	60 (11)
Some secondary	167 (54)	126 (54)	293 (54)
Completed secondary/any tertiary	107 (35)	84 (36)	191 (35)
Employed	61 (20)	49 (21)	110 (20)
Average household income			
<R1,000/month	150 (49)	83 (35)	233 (43)
R1,000–R5,000/month	125 (40)	114 (49)	239 (44)
>R5,000/month	34 (11)	38 (16)	72 (13)
SES quartile			
Lowest SES	110 (36)	56 (24)	166 (31)
Low-moderate SES	80 (26)	59 (25)	139 (26)
Moderate-high SES	70 (23)	61 (26)	131 (24)
Highest SES	49 (16)	59 (25)	108 (20)
HIV infected	113 (37)	8 (3)	121 (22)
Median recent life events [IQR]	1 [0, 2]	2 [1, 4]	1 [0, 3]
Lifetime tobacco use	15 (5)	162 (69)	177 (33)
Antenatal tobacco use	6 (2)	110 (46)	113 (21)
Lifetime alcohol use	31 (10)	156 (66)	187 (34)
Antenatal alcohol use	4 (1)	14 (6)	18 (3)
Depression—above threshold (BDI)	66 (21)	53 (23)	119 (22)
Depression—above threshold (EPDS)	80 (26)	65 (28)	145 (27)
Psychological distress—above threshold (SRQ-20)	52 (17)	56 (24)	108 (20)

### Psychosocial risk factors

Although the median IQR score for the measure of exposure to stressful life events in the past 12 months was 1 (0; 3), psychological distress (as assessed by the SRQ) was reported in 20% of participants, and approximately a quarter was found to score above threshold on measures of depression (BDI: 22%, EPDS: 27%; Table 1). Lifetime tobacco use was reported in a third of participants, with approximately 20% reporting smoking cigarettes during pregnancy. Although approximately a third of the sample also reported lifetime alcohol use, only 3% self-reported antenatal use. It is noteworthy that a site-specific difference was found across most psychosocial parameters. For example, 69% of participants at TC Newman reported lifetime tobacco use, compared with only 5% at Mbekweni, whereas 46% smoked cigarettes during pregnancy (versus 2% at Mbekweni). Similarly, 66% at TC Newman reported lifetime alcohol consumption and 6% reported antenatal consumption, whereas only 10 and 1% reported lifetime and prenatal use, respectively, at Mbekweni (Table 1).

### Trauma exposure and PTSD—prevalence and risk factors

Approximately a third of the study sample reported a history of childhood trauma (34%) and of exposure to recent (past-year) IPV (32%) (Table 2). Again, a notable site-specific difference was evident, with 43% of participants (TC Newman) versus 28% (Mbekweni) having experienced childhood trauma; and 41% (TC Newman) versus 25% (Mbekweni) reporting past-year IPV exposure. Lifetime trauma exposure was reported in approximately two-thirds (67%) of the sample. The prevalence of lifetime or recurrent PTSD (within the total sample) was 19%. Notably, participants with PTSD were also significantly more likely to meet diagnostic criteria for a major depressive disorder (OR: 6.7; 95% CI: 4.1; 11.1), suicidality (OR 3.4; 95% CI: 2.1; 5.5), and alcohol dependence (OR 2.2; 95% CI: 1.0; 4.8).

**Table 2:** Maternal trauma exposure and PTSD

Variable	Mbekweni—*n* (%)	TC Newman—*n* (%)	Total—*n* (%)
Childhood trauma—above threshold	86 (28)	101 (43)	187 (34)
Recent intimate partner violence—above threshold	77 (25)	96 (41)	173 (32)
Lifetime trauma exposure	202 (65)	164 (70)	366 (67)
Lifetime or recurrent PTSD	58 (19)	48 (20)	106 (19)

Crude analyses yielded significant associations between recent IPV exposure, psychological distress, and recent stressful life events, and trauma exposure in this study sample. In a multiple logistic regression model, recent stressful life events remained significantly associated with trauma, when controlling for SES, study site, and recent IPV (Table 3).

**Table 3:** Variables associated with lifetime trauma exposure (*n*=544)

Variable	No trauma exposure—*n* (%)	Trauma exposure—*n* (%)	Unadjusted odds ratio [95% CI]	*P*	Adjusted odds ratio [95% CI]	*P*
Recruitment site						
Mbekweni	107 (35)	202 (65)	Reference		Reference	
TC Newman	71 (30)	164 (70)	1.2 [0.9, 1.8]	0.277	1.0 [0.7, 1.5]	0.974
SES quartile						
Highest SES	36 (33)	72 (67)	Reference		Reference	
Moderate-high SES	43 (33)	88 (67)	1.0 [0.6, 1.8]	0934	1.0 [0.6, 1.8]	0.951
Low-moderate SES	48 (35)	91 (65)	0.9 [0.6, 1.6]	0.844	0.9 [0.5, 1.5]	0.666
Lowest SES	51 (31)	115 (69)	1.1 [0.7, 1.9]	0.650	1.1 [0.7, 1.9]	0.642
Recent intimate partner violence						
Below threshold	133 (36)	238 (64)	Reference		Reference	
Above threshold	45 (26)	128 (74)	1.6 [1.1, 2.4]	0.023	1.4 [0.9, 2.1]	0.107
Median recent life events [IQR]	1 [0, 2]	1 [0, 3]	1.2 [1.1, 1.3]	0.002	1.2 [1.0, 1.3]	0.008
Psychological distress (SRQ-20)				
Below threshold	159 (36)	277 (64)		
Above threshold	19 (18)	89 (82)	2.7 [1.6, 4.6]	<0.001

In a second crude analysis restricted to trauma-exposed participants only (*n*=366), PTSD was significantly more likely to occur among participants reporting lower levels of education, childhood trauma, recent stressful life events, depression (BDI and EPDS), and psychological distress (Table 4). In a multiple logistic regression model restricted to this traumatized subsample, childhood trauma and recent stressful life events remained significantly associated with PTSD, when controlling for SES and study site.

**Table 4:** Variables associated with lifetime/recurrent PTSD among trauma-exposed participants (*n=*366)

Variable	No PTSD—*n* (%)	PTSD—*n* (%)	Unadjusted odds ratio [95% CI]	*P*	Adjusted odds ratio [95% CI]	*P*
Recruitment site						
Mbekweni	144 (71)	58 (29)	Reference		Reference	
TC Newman	116 (71)	48 (29)	1.0 [0.7, 1.6]	0.907	0.7 [0.4, 1.1]	0.113
SES quartile						
Highest SES	54 (75)	18 (25)	Reference		Reference	
Moderate-high SES	60 (68)	28 (32)	1.4 [0.7, 2.8]	0.344	1.4 [0.7, 2.9]	0.349
Low-moderate SES	56 (62)	35 (38)	1.9 [0.9, 3.7]	0.070	1.6 [0.8, 3.2]	0.183
Lowest SES	90 (78)	25 (22)	0.8 [0.4, 1.7]	0.606	0.7 [0.3, 1.5]	0.354
Education						
Completed secondary/any tertiary	87 (72)	34 (28)	Reference			
Some secondary	153 (74)	54 (26)	0.9 [0.5, 1.5]	0.692		
Primary	20 (53)	18 (47)	2.3 [1.1, 4.9]	0.029		
Childhood trauma						
Below threshold	175 (76)	56 (24)	Reference		Reference	
Above threshold	85 (63)	50 (37)	1.8 [1.2, 2.9]	0.010	1.8 [1.1, 2.9]	0.029
Median recent life events [IQR]	1 [0, 3]	2 [0, 4]	1.2 [1.1, 1.3]	0.002	1.2 [1.0, 1.3]	0.008
Depression (BDI)						
Below threshold	207 (74)	73 (26)	Reference			
Above threshold	53 (62)	33 (38)	1.8 [1.1, 2.9]	0.029		
Depression (EPDS)						
Below threshold	197 (74)	68 (26)	Reference			
Above threshold	63 (62)	38 (38)	1.7 [1.1, 2.8]	0.025		
Psychological distress (SRQ-20)						
Below threshold	212 (77)	65 (23)	Reference			
Above threshold	48 (54)	41 (46)	2.8 [1.7, 4.6]	<0.001		

### Infant birth outcomes

Fifteen percent of infants in this study sample were born preterm. The median (IQR) birthweight was 3,080 (2,730; 3,420) g, and the median (IQR) head circumference at birth was 34 (33; 35) cm. On average, infants had a median WAZ of −0.5 (−1.3; 0.1) and a median HCAZ of −0.5 (−1.2, 0.2) (Table 5). Notable site-specific differences were also observed across certain birth outcomes. For example, the median (IQR) birthweight for infants at TC Newman (2,960 (2,610; 3,350) g) was lower than at Mbekweni (3,170 (2,830; 3,440) g). Furthermore, when dichotomizing birth outcomes, it was found that the prevalence of LBW at TC Newman (19%) was higher than at Mbekweni (11%). Similarly, there were differences in dichotomized WAZ and HCAZ scores, with the prevalence of low WAZ and HCAZ at TC Newman (11 and 12%, respectively) higher than at Mbekweni (6 and 8%). A higher proportion of infants was also born SGA at TC Newman (31%) than at Mbekweni (22%).

**Table 5:** Infant birth outcomes

Variable	Mbekweni—*n* (%)	TC Newman—*n* (%)	Total—*n* (%)
Number of infants; sets of twins	311 (57); 2	235 (43); 0	546
Female	163 (52)	107 (46)	270 (49)
Median gestation at delivery [IQR]	39 [38; 40]	39 [38, 40]	39 [38, 40]
Preterm birth (<37 weeks)	51 (16)	32 (14)	83 (15)
Median weight in grams [IQR]	3170 [2,830, 3,440]	2,960 [2,610, 3,350]	3,080 [2,730, 3,420]
Low birthweight (<2,500 g)	34 (11)	45 (19)	79 (14)
Median WAZ^a^ [IQR]	−0.4 [−1.2, 0.3]	−0.8 [−1.5, −0.1]	−0.5 [−1.3, 0.1]
Low WAZ (WAZ of −2 or below)	19 (6)	27 (11)	46 (8)
Small-for-gestational-age (SGA)	67 (22)	74 (31)	141 (26)
Median head circumference in cm [IQR]	34 [33, 35]	34 [32, 34]	34 [33, 35]
Median HCAZ^b^ [IQR]	−0.3 [−1.1, 0.4]	−0.6 [−1.3, 0.1]	−0.5 [−1.2, 0.2]
Low HCAZ (HCAZ of −2 or below)	25 (8)	28 (12)	53 (10)

a Weight-for-age z-score;

b head circumference-for-age z-score.

### Association between maternal trauma, PTSD, and infant birth outcomes

No association was observed between maternal PTSD and any of the birth outcomes of interest (i.e., decreased WAZ score, decreased HCAZ score, SGA, and preterm delivery). However, when examining the association between trauma exposure and adverse birth outcomes, crude analyses revealed that maternal trauma was significantly associated with a 0.3 unit reduction in infant HCAZ scores at birth (95% CI: 0.1; 0.5) (Table 6). This association remained significant when adjusted for study site, SES, and recent life stressors (Table 6, adjusted model A) in a multivariate regression model and tended toward significance when additionally adjusted for WAZ score at birth (Table 6, adjusted model B). In this analysis, both trauma exposure and PTSD were assessed over the course of participants’ lifetimes. However, data on alcohol use and smoking were only from the antenatal maternal assessment. Thus, as alcohol consumption and smoking likely acted as mediators, rather than confounders, in the relationship between trauma, PTSD, and adverse birth outcomes, these variables were not included in the final regression models.

**Table 6:** Association between lifetime trauma exposure and infant HCAZ^a^ at birth, with and without adjustment for infant WAZ^b^ at birth

Variable	Median HCAZ [IQR]	Unadjusted regression coefficient [95% CI]	*P*	(A) Model without adjustment for WAZ: Regression coefficient [95% CI]	*P*	(B) Model with adjustment for WAZ: Regression coefficient [95% CI]	*P*
Recruitment site							
Mbekweni	−0.3 [−1.1, 0.4]	Reference		Reference		Reference	
TC Newman	−0.6 [−1.3, 0.1]	−0.3 [−0.6, −0.1]	<0.001	−0.3 [−0.5, −0.1]	0.013	−0.02 [−0.2, 0.2]	0.780
SES quartile							
Highest SES	−0.3 [−1.0, 0.2]	Reference		Reference		Reference	
Moderate-high SES	−0.5 [−1.3, 0.1]	−0.2 [−0.5, 0.1]	0.173	−0.2 [−0.5, 0.1]	0.127	−0.04 [−0.3, 0.2]	0.755
Low-moderate SES	−0.5 [−1.5, 0.3]	−0.2 [−-0.5, 0.1]	0.241	−0.2 [−0.5, 0.1]	0.195	−0.005 [−0.2, 0.2]	0.970
Lowest SES	−0.4 [−1.2, 0.3]	−0.04 [−0.3, 0.3]	0.799	−0.1 [−0.4, 0.2]	0.516	0.1 [−0.1, 0.4]	0.347
Antenatal tobacco use							
Below threshold	−0.4 [−1.2, 0.4]	Reference					
Above threshold	−0.6 [−1.3, 0.1]	−0.3 [−0.6, −0.1]	0.017				
Antenatal alcohol use							
Below threshold	−0.4 [−1.2, 0.3]	Reference					
Above threshold	−1.3 [−1.8, –0.6]	−0.9 [−1.4, −0.3]	0.002				
Intimate partner violence (IPV) exposure							
No recent IPV	−0.3 [−1.2, 0.4]	Reference					
Any recent IPV	−0.6 [−1.3, 0.1]	−0.3 [−0.5, −0.1]	0.006				
Recent life events		−0.1 [−0.1, −0.03]	0.001	−0.1 [−0.1, −0.0003]	0.049	−0.03 [−0.1, 0.01]	0.150
Infant WAZ		0.7 [0.6, 0.8]	<0.001			0.7 [0.6, 0.8]	<0.001
Trauma exposure							
No exposure	−0.2 [−1.2, 0.5]	Reference		Reference		Reference	
Trauma exposure	−0.6 [−1.3, 0.1]	−0.3 [–0.5, −0.1]	0.009	−0.2 [−0.5, −0.03]	0.026	−0.2 [−0.3, 0.03]	0.054

a Head circumference-for-age z-score;

b Weight-for-age z-score.

## Discussion and conclusions

The key findings of this exploratory study of maternal psychological trauma, PTSD, and associated birth outcomes were (1) the majority of women had experienced at least one lifetime traumatic event, including childhood abuse and/or IPV, with a notable prevalence of PTSD; (2) childhood trauma and recent stressful life events were significantly associated with PTSD, when controlling for SES and study site; (3) although infant anthropometric markers at birth were within the WHO “normal” range, there was a notable site-specific difference across a number of parameters, with infants born to mothers from TC Newman fairing more poorly overall; and (4) no associations were observed between maternal PTSD and subsequent birth outcomes. However, our findings suggest that maternal psychological trauma may be significantly associated with a reduction in head circumference z-score at birth, when controlling for a number of potentially confounding variables.

The high trauma burden in our study sample is largely in line with the local and international literature. For example, although the National Comorbidity Survey reported that approximately 60% of men and 50% of women had been exposed to at least one traumatic event (Kessler et al., 1995), findings from the SASH indicated that nearly 75% of South Africans experienced some trauma during their lifetimes (Williams et al., 2007). It is noteworthy, however, that the prevalence of PTSD in our sample (19%) was far higher than has been reported previously (e.g., Herman et al., 2009; Kessler et al., 1995). This may be due to a number of interrelated factors. First, ours is a particularly at-risk population, with the prevalence of depression and substance use notably higher than those in the SASH (Herman et al., 2009). Both factors may predispose individuals to the development of PTSD following trauma exposure. Second, our study sample comprises only women of reproductive age. PTSD exhibits a significant gender bias, which may be related to environmental or neurobiological factors. Third, the high level of internal migration and urban transition found in our study sample imposes an additive risk. In addition to socioeconomic inequity, exposure to xenophobic behaviors (e.g., Harris, 2002), employment instability, and exclusion from citizenship rights may place additional stress on migrant households and individuals (Tacoli et al., 2015).

Childhood trauma and stressful life events were found to be significantly associated with PTSD. The role of early negative life events in precipitating or exacerbating psychological disorders in adulthood has been well documented (Yehuda, Spertus, & Golier, 2001). For example, in her study of 1,196 victims of substantiated child abuse and neglect from 1967 to 1971 in a midwestern metropolitan county area, United States, Widom (1999) reported that childhood victimization was associated with an increased risk of lifetime and current PTSD, with 37.5% of victims of childhood sexual abuse, 32.7% of victims of childhood physical abuse, and 30.6% of victims of childhood neglect meeting the DSM criteria for lifetime PTSD. This association may be underpinned by revictimization of the survivors of childhood maltreatment (e.g., Messman-Moore & Long, 2003), as well as by neurobiological alterations such as hyperactive stress reactivity and exaggerated hypothalamic-pituitary-adrenal (HPA) axis functioning (Heim & Nemeroff, 2001; Heim et al., 2001). Furthermore, in two meta-analyses of the predictors of PTSD in adults (Brewin, Andrews, & Valentine, 2000; Ozer, Best, Lipsey, & Weiss, 2003), it was found that prior trauma and additional life stress significantly increased the risk of developing this disorder.

In our sample, measures of infant anthropometry at birth were found to be within the normal range according to the WHO child growth standards (WHO, 2014). This may be accounted for by the relatively high level of education among our maternal study population, as well as by the perinatal care and health promotion inherent in a longitudinal study such as ours. However, the site-specific difference in birth anthropometry (with poorer outcomes at TC Newman) may be attributable to the discordant risk factor profile between the two sites (see also Koen et al., 2014.) For example, the prevalence of maternal lifetime and prenatal tobacco/alcohol use, as well as of childhood trauma and past-year IPV, were all found to be higher at TC Newman than at Mbekweni, which is in line with the poorer birth outcomes at TC Newman.

In contrast to a notable evidence base of the adverse effects of prenatal psychological trauma on birth outcomes, neither maternal trauma nor PTSD was significantly associated with a reduction in most anthropometric measures, or with a shortened gestation in these data. In their recent systematic review of 49 peer-reviewed studies assessing the effect of disasters on pregnancy outcomes, maternal mental health, and child development, Harville, Xiong, and Buekens (2010) concluded that a major concern for pregnant women exposed to disaster relates to decreased fetal growth, particularly in the most directly exposed women. Similarly, there is a large body of work documenting the association between maternal stress or distress, and outcomes such as LBW and preterm delivery. In their prospective, population-based study of 5,872 women with singleton pregnancies attending an antenatal care clinic in Denmark, Hedegaard, Henriksen, Sabroe, and Secher (1993) reported that—compared to low psychological distress—the relative risk of preterm delivery for moderate distress during late pregnancy was 1.22 (95% CI 0.84; 1.79), and for high distress it was 1.75 (95% CI 1.20; 2.54). Furthermore, there is evidence that maternal exposure to prenatal life event stress may be associated with a notable decrease in infant birthweight, independent of biomedical risk (Wadhwa, Sandman, Porto, Dunkel-Schetter, & Garite, 1993). In their longitudinal cohort study of 1,100 pregnant women recruited from prenatal clinics in inner-city New Haven, Connecticut, Rogal et al. (2007) also reported that preterm delivery was nearly three times more likely in participants with PTSD versus those without (OR=2.72, 95% CI: 0.91, 8.14).

The discrepancy between our study findings and this compelling body of published work may be due to our limited power to detect smaller effect sizes; to our use of different assessment tools, each with measurement biases that may have reduced our ability to detect associations; to our LMIC study setting (in contrast to the high-income populations investigated in most prior work); or to the temporality of risk factors (e.g., “recent,” or past-year trauma may have pre-dated pregnancy). Furthermore, it may be that diverse maternal risk factors are associated with different effects on fetal growth and intrauterine development. For example, our study group has previously demonstrated a significant association between maternal exposure to IPV and delivery of a LBW infant in the Drakenstein cohort (Koen et al., 2014). Although we did not investigate associations of low infant birthweight in the current analysis, maternal trauma was found to be significantly associated with reduced HCAZ at birth in our final multivariate model. This is in line with one study (Engel, Berkowitz, Wolff, & Yehuda, 2005) which found that women with posttraumatic stress symptomatology resulting from the World Trade Center attacks were more likely to deliver infants with reduced head circumference at birth (beta=−0.07, SE=0.03, *P*=0.01). It may also be seen as complementary to another prior study by our group, demonstrating that maternal depression is significantly associated with reduced HCAZ scores in the Drakenstein cohort, when adjusted for study site, SES, and recent stressful life events (Brittain et al., 2015). Taken together, these findings suggest that different maternal risk factors, measured by a range of assessment tools (each with unique biases) and acting through diverse mediators, may contribute to the heterogeneity of observed associations with infant birth outcomes in this vulnerable study population.

In fact, it is likely that a number of biological and behavioral mediators underlie the association between maternal psychological trauma and reduced fetal head growth. From a neurobiological perspective, it is widely hypothesized that stress activates both the HPA axis and the sympathetic nervous system (SNS) (e.g., Vermetten & Bremner, 2002). Thus, elevated levels of corticotrophin releasing hormone, adrenocorticotropin hormone, and cortisol (consistent with a hyperactive stress response) may contribute to fetal growth restriction, as has been documented (e.g., Challis et al., 2001; Wadhwa et al., 2004). Furthermore, abnormally elevated levels of adrenaline and noradrenaline (characteristic of SNS hyperactivation) may be associated with increased uterine artery resistance, likely mediated by sympathetic vasoconstriction (Teixeira, Fisk, & Glover, 1999). Intrauterine growth restriction may in turn result from impaired transplacental blood flow (Bower, Bewley, & Campbell, 1993). Behavioral mediators (such as smoking and alcohol consumption) and other health condition variables have also been found to be associated strongly with maternal trauma experiences and are likely to influence birth outcomes. For example, cigarette smoking has been shown to be a significant mediator in the relationship between maternal exposure to recent IPV and delivery of a LBW infant (Kearney, Munro, Kelly, & Hawkins, 2004). Health conditions, such as maternal psychopathology, obesity, metabolic syndrome, and HIV infection, have also emerged as potential mediators in prior work (see Campbell, 2002; Ferri et al., 2007; Stene, Jacobsen, Dyb, Tverdal, & Schei, 2013), as have social and family support networks (e.g., Ozbay, Fitterling, Charney, & Southwick, 2008; Ozbay et al., 2007). Thus, there is likely a diverse and interacting range of mediators linking maternal trauma exposure to adverse birth outcomes, and further work to delineate these variables is warranted.

Limitations of the current study include potential recall bias resulting from self-reported maternal data and limited generalizability of findings due to the relative homogeneity of the study sample. Although clinician-administered assessment tools (i.e., the MINI) were used to supplement self-reported findings, some of these assessments were completed at a timepoint after the index infants’ birth, thus potentially complicating the temporality of the associations. Furthermore, as this study was conducted in a peri-urban region of low SES, the study sample may not be generally representative of South Africa. Another major limitation of the current study is the strong likelihood of unmeasured and residual confounding. For example, although we adjusted for the effect of study site and SES in our analysis, the standardized conceptualization and accurate measurement of the latter variable remains highly challenging (Ndhlovu & Khalema, 2015; Oakes & Rossi, 2003), and even good measures are likely to retain some residual confounding (Kaufman, Cooper, & McGee, 1997). Finally, only pregnant women accessing antenatal care who consented to participate were recruited. Data from those not seeking care and those refusing to participate in the study were not included. Thus, sampling bias may have occurred, as the risk profile and birth outcomes of enrolled women may have differed from those not enrolled.

Despite its limitations, our study constitutes a novel exploratory investigation of the prevalence of, and risk factors for, psychological trauma and PTSD during pregnancy, as well as associated birth outcomes, in a LMIC population in transition. However, given the high prevalence of PTSD found in our sample, as well as the methodological shortcomings of the current study, there is a clear need for further research in settings such as ours, focused on the association between stressful life events, traumatic exposure, and consequent PTSD. Adequate detection and appropriate treatment of trauma-exposed individuals in the pre- and peripartum period would be helpful to curb the detrimental effects on mother and child. Potential targets of such interventions may include antenatal substance use, psychosocial stress, and other mediators of the association between maternal trauma and adverse birth outcomes. Given the complexity of these causal pathways, intervention strategies may well require a multidimensional approach. Longitudinal follow-up of maternal psychological risk, mother–child attachment, and measures of developmental and child mental health will be important to evaluate the long-term functional effects of maternal trauma exposure. In the future, further work in this field would be useful to extend our findings and to delineate causal pathways and neurobiological mechanisms.
